# Goats as Valuable Animal Model to Test the Targeted Glutamate Supplementation upon Antral Follicle Number, Ovulation Rate, and LH-Pulsatility

**DOI:** 10.3390/biology11071015

**Published:** 2022-07-06

**Authors:** Luis A. Luna-García, César A. Meza-Herrera, Carlos C. Pérez-Marín, Rebeca Corona, Juan R. Luna-Orozco, Francisco G. Véliz-Deras, Ramón Delgado-Gonzalez, Rafael Rodriguez-Venegas, Cesar A. Rosales-Nieto, Jorge A. Bustamante-Andrade, Ulises N. Gutierrez-Guzman

**Affiliations:** 1 Universidad Autónoma Chapingo, Unidad Regional Universitaria de Zonas Áridas, Bermejillo 35230, Durango, Mexico; tonylunamvz@gmail.com; 2 Departamento de Medicina y Cirugía Animal, Campus Rabanales, Universidad de Córdoba, 14014 Córdoba, Spain; pv2pemac@uco.es; 3 Departamento de Neurobiología Celular y Molecular, Laboratorio de Neuroanatomía Funcional y Neuroendocrinología, Instituto de Neurobiología, UNAM, Querétaro 76230, Mexico; rebecgc@gmail.com; 4 Centro de Bachillerato Tecnológico Agropecuario No. 1, Torreón 27000, Coahuila, Mexico; jlunaorozco@yahoo.com.mx; 5 Universidad Autónoma Agraria Antonio Narro, Unidad Laguna, Torreón 27054, Coahuila, Mexico; velizderas@gmail.com (F.G.V.-D.); raldego@gmail.com (R.D.-G.); rafar.v.v@gmail.com (R.R.-V.); 6 Facultad de Agronomía y Veterinaria, Universidad Autónoma de San Luis Potosí, San Luis Potosí 78321, Mexico; nieto_cesar@hotmail.com; 7 Facultad de Agricultura y Zootecnia, Universidad Juárez del Estado de Durango, Venecia Durango 35111, Mexico; abaj_86@hotmail.com (J.A.B.-A.); ulisesnoelg@yahoo.com.mx (U.N.G.-G.)

**Keywords:** goats, animal models, glutamate, LH, ovarian function, translational models

## Abstract

**Simple Summary:**

Exogenous glutamate administration to yearling-anestrous goats enhanced not only ovarian function (i.e., ovulation rate and antral follicle number) but also denoted undoubted effects at the hypothalamic-pituitary level, augmenting LH pulsatility. Our results show not only the use of glutamate as a clean alternative but also as an interesting reproductive strategy to amplify ovarian function, considering goats as an animal model. While this should enlarge the possibility of escalating our knowledge regarding the hypothalamic-pituitary-gonadal modulation by glutamate, such research outcomes may also embrace potential translational implications.

**Abstract:**

The potential effect of intravenous administration of glutamate on the ovarian activity and the LH secretion pattern, considering the anestrous yearling goat as an animal model, were assessed. In late April, yearling goats (*n* = 20) were randomly assigned to either (1) Glutamate supplemented (GLUT; n = 10, Live Weight (LW) = 29.6 ± 1.02 kg, Body Condition (BCS) = 3.4 ± 0.2 units; i.v. supplemented with 7 mg GLUT kg^−1^ LW) or (2) Non-supplemented (CONT; *n* = 10; LW = 29.2 ± 1.07 kg, BCS = 3.5 ± 0.2 units; i.v. saline). The oats were estrus-synchronized; blood sampling (6 h × 15 min) was carried out for LH quantification. Response variables included pulsatility (PULSE), time to first pulse (TTFP), amplitude (AMPL), nadir (NAD), and area under the curve (AUC) of LH. Ovaries were ultra-sonographically scanned to assess ovulation rate (OR), number of antral follicles (AF), and total ovarian activity (TOA = OR + AF). LH-PULSE was quantified with the Munro algorithm; significant treatment x time interactions were evaluated across time. The variables LW and BCS did not differ (*p* > 0.05) between the experimental groups. Nevertheless, OR (1.77 vs. 0.87 ± 0.20 units), TOA (4.11 vs. 1.87 ± 0.47 units) and LH-PULSE (5.0 vs. 2.2 pulses 6 h-1) favored (*p* < 0.05) to the GLUT group. Our results reveal that targeted glutamate supplementation, the main central nervous system neurotransmitter, arose as an interesting strategy to enhance the hypothalamic–hypophyseal–ovarian response considering the anestrous-yearling goat as an animal model, with thought-provoking while promising translational applications.

## 1. Introduction

In goats, the seasonal variations in ovarian activity are the direct result of changes in the release of the gonadotropin-releasing hormone (GnRH) secretion, mainly exerted through the changing actions of the photoperiod [1,2]. Indeed, the photoperiod is the main environmental cue that modulates reproductive seasonality [3,4], altering the negative feedback exerted by estradiol (E2) upon the luteinizing hormone (LH) secretion [5,6]. The increased negative feedback of E2 on LH secretion has been accepted as the mechanism responsible for seasonal reproduction in most small ruminants [5,6]. In addition, glutamate (GLUT) has been recognized as the main, fast-acting excitatory neurotransmitter of the central nervous system (CNS), regulating most of the excitatory synaptic transmissions in the brain, while also involved in several biological processes [7]. GLUT-receptors have been localized in the diverse hypothalamic nuclei, some of which are key to the reproductive and neuroendocrine functions [8]. Moreover, the functional glutamatergic systems have been demonstrated in non-neural peripheral tissues, such as the heart, kidney, and gonads [7,8]. At a central level, the glutamatergic systems (i.e., ligand-receptors), have been involved in the control of pulsatile GnRH secretion, and the pre-ovulatory surge of gonadotropins (LH and FSH) [9]. Interestingly, the glutamatergic neurons that have been associated in the control of the GnRH neurons are also responsive to kisspeptin, an essential peptide in the regulation of the hypothalamic–pituitary–gonad (HPG) axis, promoting an increase in GnRH pulses [8,9,10]. While domestic sheep and goats have shown great potential as large animal models, sheep have been mainly used to perform preclinical and translational studies in reproductive research, while goats have been used in a more limited fashion [10]. We hypothesized a positive effect of the intravenous GLUT-supplementation upon ovarian function through an improved release profile of LH, considering the anestrous-yearling goat as the animal model. Thus, this study was designed to solve such a working hypothesis.

## 2. Materials and Methods

### 2.1. Location, Ethical-Welfare Issues, and Animal Management

The present study was carried out in northern Mexico (26° N, 103° W; 1120 m), in an intensive, commercial goat production system. Yearling anestrous Alpine-Saanen-Nubian × Criollo goats (*n* = 20), with an average live weight (LW) of 29.17 ± 1.02 kg and body condition score (BCS) of 3.45 ± 1.02 units, were involved. The experiment was conducted along the natural anestrous season, during April and May, i.e., under long-day photoperiodic conditions. The LW and BCS (from 1 = emaciated to 5 = obese) were weekly recorded before feeding by an experienced technician. All of the experimental procedures were completed following the recommendations for ethical use, care, and welfare of animals in research at global [11] and national [12] levels, and they were institutionally authorized (UACH-DGIP-REBIZA-IBIODEZA/15-510-400-2).

### 2.2. Experimental Design

At the end of April, the animals were individually housed in pens and they were randomly assigned to two experimental groups: (1) Glutamate (GLUT; *n* = 10) and (2) Control (CONT; *n* = 10). The LW and BCS were similar in both GLUT (29.1 ± 1.02 kg, 3.4 ± 0.2 units) and CONT (29.2 ± 1.07 kg, 3.5 ± 0.2 units) groups. All of the animals received a basal diet twice per day (07:00 and 16:00), consisting of alfalfa hay (14% crude protein, 4.7 MJ/kg), corn grain (11.2% crude protein, 9.9 MJ/kg), and corn silage (8.1% crude protein, 6.7 MJ/kg), balanced to cover their net energy requirements for maintenance [13]. Additionally, the GLUT-goats were supplemented every third day during the experimental period, with an intravenous injection of glutamate (L-glutamate, Merck-C5H9NO4-art-101791; from day 34 pre-estrus to day 17 post-estrus). Animals had ad libitum water access and shaded areas. The composition values of the components of the basal diet (Dry Matter (DM)% basis) were obtained from representative samples taken throughout the experimental period and analyzed based on the formerly defined techniques [14].

### 2.3. Estrus Synchronization, Blood Sampling, and LH Determinations

The estrus synchronization was initiated 23 days after the beginning of the experiment. Intravaginal progestogen-impregnated sponges containing 45 mg of fluorogestone acetate (Chronogest^®^; Intervet International B.V., Boxmeer, Holland) were inserted for 10 days; one day before the sponge withdrawal, the goats received an i.m. dose of 75 μg of D-cloprostenol (Prosolvin-C^®^, Intervet International B.V., Boxmeer, Holland). Five goats per group were randomly selected 24 h after the sponges’ withdrawal (−1 day); blood samples were collected at 3 h after the morning feeding. A volume of 10 mL of blood was collected every 15 min for 6 h by jugular venipuncture using sterile vacuum tubes (Corvac; Kendall Health Care, St. Louis, MO, USA) and allowed to clot at room temperature for 30 min. The blood was centrifuged (1500× *g*, 15 min) to obtain serum, and then it was decanted and stored into polypropylene microtubes (Axygen Scientific, Union City, CA, USA) at −20 °C until assayed. The peripheral serum LH concentrations were measured in duplicate by radioimmunoassay, as previously described [15]. The assay sensitivity was 0.2 ng/mL and intra-assay variation coefficient for LH quantification was 10%; the Munro algorithm was used to identify the LH pulses [16]. While the LH basal levels were quantified as the average of the lowest obtained values [17,18], the area under the curve (AUC) of LH was also determined.

### 2.4. Ultrasonographic Evaluation of Ovarian Activity

**Figure 1:**
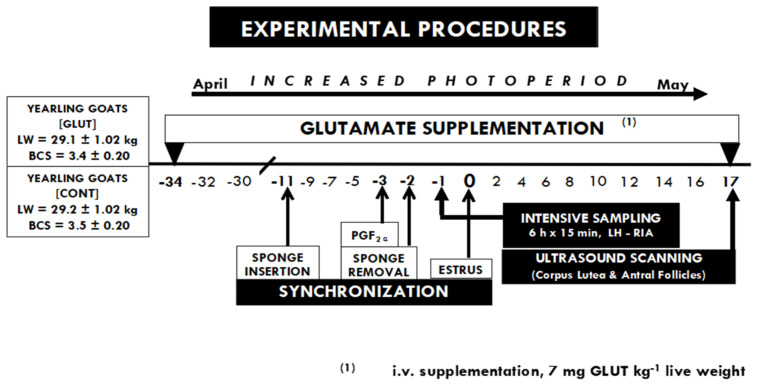
Time-line of activities performed during the experiment, including the estrous synchronization protocol and the targeted glutamate i.v.—supplementation every third day along with the experimental period, (L-glutamate, from day 34 pre-estrus to day 17 post-estrus). Blood sampling (every 15 min × 6 h) for LH quantification was performed 24 h prior to the estrus day (day 0). Later, ovarian ultrasonographic assessment was carried out on day 17 post-estrus to study the relationship between the TOA and LH secretion pattern. All the experimental units had ad libitum water access and shaded areas in each pen.

On day 17 post-estrus (coincident with the end of the luteal phase), an ultrasonographic assessment was carried out by a qualified operator to monitor the ovarian activity, using an ultrasound scanner (Toshiba Medical Systems Ltd., Crawley, UK), equipped with a 7.5 MHz linear-array transducer. The ovaries were scanned to record the number of corpus luteum (which indicate the ovulation rate, OR) and the antral follicles (AF; those ≥ 5 mm) [19]. Then, the total ovarian activity (TOA) was considered as AF + OR, recorded in both of the ovaries in each animal within the experimental group. The main activities performed during the experimental period are depicted in Figure 1.

### 2.5. Statistical Analyses

The variables LW, BCS, OR, TOA, and serum LH concentrations were evaluated using the PROC-MIXED of SAS (SAS Institute Inc., Cary, NC, USA), for repeated measures across time in the same animal. Each goat within the experimental group was defined as the experimental unit. The experimental treatment (i.e., GLUT or CONT) and the sampling day (i.e., Time) were analyzed using mixed linear model procedures and the estimation technique of restricted maximum likelihood (PROC MIXED). While time was considered as the repeated measure, the treated goats were defined as the repeated subject and regarded as the random error term [20]. In the case of mean significant differences of the analyzed response variables across time, these were solved through the LSMEANS-LSD option of PROC GLM. Since the LH pulse frequency showed a non-parametric distribution, a Kruskal–Wallis test was used to analyze the variable LH-PULSE. The response variables were evaluated for normality using the Shapiro–Wilk test, and log^10^, transformed for basal and mean LH concentrations as well as for LH pulse amplitude. When a significant effect of the treatment x time interaction occurred, the data were compared across time. Pearson’s correlations were used to check the associations among the variables LW, BCS, and OR. Non-transformed data are shown and expressed as least-square means ± standard error (SE). All of the statistical analyses were carried out using the procedures and options of SAS (SAS Inst. Inc., V9.1, Cary, NC, USA); significant differences between means were set at *p* < 0.05.

## 3. Results

**Figure 2:**
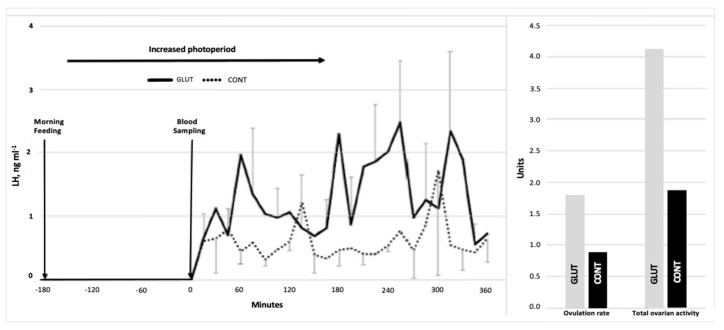
Serum LH concentrations (ng/mL) across time (**left**), and OR (units) and TOA (units) (**right**) in glutamate-supplemented (GLUT) and non-supplemented (CONT) goats.

The comparison of LW and BCS at the beginning (29.4 ± 1.02 kg and 3.4 ± 0.17) and the end of the experimental period (35.13 ± 1.07 kg and 3.4 ± 0.2) did not differ between the experimental groups (*p* > 0.05). Interestingly, however, the differences (*p* < 0.05) were observed for OR and TOA (1.77 vs. 0.87 ± 0.20) and (4.11 vs. 1.87 ± 0.47), respectively, favoring the GLUT-treated group. Moreover, an increased LH-PULSE (5.0 vs. 2.2 pulses 6 h^−1^, *p* < 0.05) also favored the GLUT-treated group (Table 1). The LH-pattern release across time, along with OR and TOA, are shown in Figure 2. Further, positive correlations occurred between LW1 and BCS1 (r^2^ = 0.71; *p* < 0.01), BCS and TOA (r^2^ = 0.7, *p* < 0.05), AF and OR (r^2^ = 0.61, *p* = 0.01), and OR and TOA (r^2^ = 0.87, *p* = 0.001).

## 4. Discussion

The results obtained backs both our working hypothesis as well as our animal model; the intravenous supply of glutamate to anestrous-yearling goats as an animal model endorsed the increases in both ovulation rate and total ovarian activity, while it simultaneously generated rises in LH pulsatility, considering the anestrous-yearling goat as an animal model. Whereas, no differences occurred between the experimental groups regarding LW and BCS, in addition, the response variables time of the first LH pulse, the LH amplitude, LH-nadir, and LH-AUC were also not different between the groups. This neuroendocrine and physiologic scenario suggests that the glutamate supplementation to yearling-anestrous goats exerted a positive effect upon the hypothalamic centers responsible for GnRH, and, thereafter, upon the anterior pituitary, augmenting the LH pulsatility. The specific site of action of glutamate along the hypothalamic–pituitary–ovarian continuum, as well as its role within the neuronal pathway responsible for the inhibitory effects of E2 during seasonal anestrus in goats, await to be elucidated.

The ability of E2 to inhibit the release of GnRH and LH is the mechanism responsible for the variation in ovarian activity during anestrous [21]. Such E2-negative action upon the LH-pulse generator is facilitated by a group of neurons located in the retrochiasmatic area of the hypothalamus, known as the A15 dopaminergic neurons [22]. These neurons release dopamine and through the dopamine type 2 receptor (D2R), which are present in the kisspeptinergenic neurons of the arcuate nucleus (ARC), inhibit the release of the peptide kisspeptin, resulting in a decrease in GnRH and LH pulses during the anestrous season [5]. Most of the brain cells use glutamate as the main fast-acting excitatory neurotransmitter [7]. This neuroexcitatory amino acid and its receptors are distributed throughout the CNS and diverse parts of the body, including the ovary [23]. Likewise, glutamatergic and kisspeptinergenic neurons have been involved in different physiological processes, including reproductive ones, such as the activation of the hypothalamic axis, particularly in the production of the preovulatory GnRH-LH peak [24].

The GnRH neurons are stimulated by glutamate through the activation of the ionotropic receptors, as N-methyl-D-aspartate (NMDA), and amino-3-hydroxyl-5-methyl-4-isoxazole-propionate (AMPA) [25]. In goats, the glutamate administration has shown positive effects upon diverse reproductive outcomes. The previous studies of our group have exposed that the glutamate supply diminishes the E2-negative feedback exerted on the hypothalamus–pituitary axis, modulating ovarian function and metabolic hormone synthesis [10]. The results of our study suggest that the responses generated by glutamate administration could have acted upon the ionotropic ovarian receptors [26,27], increasing the ovulatory response. Besides, the glutamate supply may have promoted the increase in metabolic hormones and growth factors key to ovarian function, increasing follicular cell proliferation, and augmenting E2 synthesis [10]. Such a neurophysiological and endocrine scenario should have promoted a positive E2-effect upon the hypothalamus–pituitary axis, increasing the GnRH-LH pulsatility, and activating, in turn, ovarian activity.

Indeed, these results suggest that, in yearling-anestrous goats supplemented with intravenous glutamate, the inhibitory E2-feedback upon the hypothalamus–pituitary axis would be considerably diminished. Such an administration of glutamate probably acted somewhere along the neuronal continuum responsible for blocking GnRH secretion during the natural anestrous season, decreasing the action of dopamine on the kisspeptin-producing neurons, while enhancing the activation of the hypothalamic centers responsible for GnRH synthesis and secretion. The last, in turn, may have increased the LH-pulse, while augmenting the ovarian activity. Whereas diverse studies support the positive effects of glutamate administration upon the reproductive outcomes in diverse species, either male [28] or female [10,24,29,30], other studies have also demonstrated an interesting interplay between glutamatergic signaling and sexual male-to-female behavior [28,31].

Some components of the rams’ sexual behavior such as ano-genital sniffing, vocalizations, mounting, intromission, and ejaculation are also observed in rodents [32,33,34]. Preceding studies of our research group demonstrated that parenteral glutamate supply enhanced male sexual behavior, both appetitive and consummatory, either in adult [32] or pubertal [33] rams. Recent reports have also shown that glutamatergic neurons in the ventral tegmental area of the lateral hypothalamus are activated by, and required for, innate defensive reactions [35]. Moreover, GnRH stimulates the LH release via glutamatergic receptors expressed in the hypothalamic kisspeptin neurons in both peripubertal [36] and adult [37] females. Such amplified glutamatergic neuron transmission effect is enhanced because of the co-expression of kisspeptin, neurokinin-B, and dynorphin, the so-called glutamatergic KNDy neurons, which drive the episodic release of GnRH [38,39]. Besides, increases in the ARC Kiss1, and Pdyn expression were positively correlated not only with gonadotropin secretion and follicular growth, but also with an augmented positive energy balance [40].

Based on the obtained research outcomes from our study, and merged with those generated by others previously discussed, a sensible question is, how to align these outcomes with a translational perspective? In humans, female infertility is one of the main public health problems worldwide [41]; whereas female infertility represents 37% of the causes in infertile couples [42], the most common factors causing women reproductive dysfunctions mainly involve ovulatory disorders (25%) and ovarian dysfunctions (50%) [43]. Such women’s reproductive failures have also been linked, unfortunately, to mental, emotional, and physical issues [44]. The clinical management of infertility has included active ovarian stimulation treatments [45] to lessen a poor ovarian response or primary ovarian insufficiency; the key aim is to diagnose and then be able to manage women’s infertility [46]. As noted, ovine and, to a lesser extent, caprine, have been used as large animal models for diverse research purposes. The use of the former ranges from the generation of therapeutic agents in the mammary glands, up to their use in human genetic disorders and regenerative medicine, as well as the edition of their genomes using CRISPR-based systems for diverse translational purposes [47]. Hence, building on the diverse research outcomes generated from the various goat research models presented along with this discussion, and considering the need to develop basic biomedical research for studying the neurophysiological functions and dysfunctions along with the hypothalamic–pituitary–ovarian axis to upgrade women’s reproductive fitness, our research outcomes undoubtedly unveiled the interesting role that goats can play as a successful experimental animal model.

## 5. Conclusions

The targeted glutamate intravenous administration to anestrous-yearling goats positively influenced not only the ovarian function (i.e., ovulation rate and antral follicle number) but also undeniably denoted effects at the hypothalamic–pituitary level (i.e., an augmented LH pulse). Such a neurophysiological complex scenario was certainly not observed in the control group. Our results show the use of glutamate to be a clean, green, and ethical alternative, substituting the use of exogenous hormones, while providing an interesting reproductive strategy to augment the activity of the hypothalamic–pituitary–ovarian continuum (HPOC). Our research outcomes should enlarge the possibility of escalating the knowledge about the action of a targeted neurotransmitter supply, such as glutamate, upon the endocrine and physiological mechanisms involved in the enhancement of the HPOC response, through the use of a goat animal model. Certainly, the research outcomes that stemmed from this study, using the goat as an experimental model, should augment the confidence in the design of biomedical reproductive studies. Besides, a potential glutamate-modulated cross-talk among the CNS and the HPOC requires clarification. Undoubtedly, the obtained results from this study await and deserve to be tested in clinical trials with potential translational applications.

## Figures and Tables

**Table 1:** Least-square means regarding LW and BCS at the onset of treatments (-initial) and at the ultrasound scanning (-ultrasound), ovulation rate (OR), total ovarian activity (TOA), and LH profile across time (pulsatility, time to first pulse, amplitude, nadir and AUC) in goats supplemented with glutamate (GLUT) and non-supplemented (CONT) groups.

	GLUT	CONT	S.E. ^1^
LW-initial (kg)	29.60 ^a^	29.24 ^a^	1.02
BCS-initial (units)	3.4 ^a^	3.5 ^a^	0.17
LW-ultrasound (kg)	35.06 ^a^	35.21 ^a^	1.07
BCS-ultrasound (units)	3.5 ^a^	3.2 ^a^	0.20
Ovulation rate (units)	1.77 ^a^	0.87 ^b^	0.20
Total ovarian activity (units)	4.11 ^a^	1.87 ^b^	0.47
LH pulsatility, pulses/6 h (units)	5.0 ^a^	2.2 ^b^	0.60
LH time to first pulse (min)	35.0 ^a^	81.0 ^a^	31.99
LH amplitude (ng)	2.35 ^a^	1.16 ^a^	0.70
LH nadir (ng)	0.43 ^a^	0.20 ^a^	0.11
LH AUC (arbitrary units)	72.6 ^a^	40.0.0 ^a^	21.5

^a,b^ Least-square-means without a common superscript, differ (*p* < 0.05) ^1^ Most conservative standard error is presented.

## Data Availability

None of the data were deposited in an official repository, yet, information can be made available upon request.
